# PaEDNet: A Robust Denoising and Classification Framework for Vibration-Based Fault Diagnosis with Measurement Noise

**DOI:** 10.3390/s26113435

**Published:** 2026-05-29

**Authors:** Xiaojing Liao, Yongwei Chi, Yu Bai, Qinya Dai, Peiyu Zhao, Na Li, Linlin Sun, Dongyang Li

**Affiliations:** 1 Advanced Institute of Information Technology, Peking University, Hangzhou 311215, China; 24s010047@stu.hit.edu.cn (X.L.);; 2 JIE Holding Group Co., Ltd., Hangzhou 311223, China; 3 College of Mechanical Engineering, Zhejiang University, Hangzhou 310028, China; 4 Hangzhou Special Equipment Inspection and Science Research Institute, Hangzhou 310051, China

**Keywords:** phase-space reconstruction, vibration imaging, expert-adaptive denoising, mixture-of-experts, DnCNN, DenseNet

## Abstract

To address the problem of fault-related structures and noise disturbances in rolling bearing vibration signals being highly coupled in the original one-dimensional signal domain under severe noise conditions, in this study, we propose a **P**hase-space **a**daptive **E**xpert **D**enoising **Net**work (PaEDNet), a robust fault diagnosis framework that integrates representation construction, adaptive restoration, and condition discrimination. Unlike existing methods that mainly enhance network modelling directly in the original signal domain, the proposed framework first constructs a spatially organised two-dimensional similarity representation through phase-space reconstruction, which further unfolds fault-related dynamic structures from temporal entanglement and provides a more suitable preliminary representation domain for subsequent restoration. On this basis, a CoPaMoE-augmented adaptive denoising module is introduced into the representation domain to improve structural restoration capability under heterogeneous noise and different local patterns. DenseNet is then employed for fault classification, thereby forming an integrated fault diagnosis framework combining representation reconstruction, noise restoration, and condition discrimination. The resulting pipeline performs end-to-end diagnosis from raw vibration signals to fault labels at inference, while training is conducted in a stage-wise manner. Experimental results derived using the two public datasets, CWRU and PU, show that the proposed method consistently outperforms multiple comparative models under different signal-to-noise ratio conditions and maintains stronger robustness in low-SNR scenarios. Under the −6 dB condition, PaEDNet achieves classification accuracies of 93.98% and 90.45% on the two datasets, respectively. Further ablation studies and expert-routing analysis demonstrate that the combination of structured representation construction and adaptive expert restoration jointly enables the improved performance of the model. In this study, we provide a new modelling perspective for the fault diagnosis of vibration signals in complex noisy environments.

## 1. Introduction

Rolling bearings are key components of rotating machinery and are among the most failure-prone parts of such tools [1,2]. Given the high sensitivity of vibration signals to operating conditions, vibration-based fault diagnosis has become an important tool for bearing health monitoring. However, under real operating conditions, multiple interference sources, including mechanical vibration, environmental noise, and electromagnetic disturbance, are often reflected as noise contamination in measured vibration signals, which can obscure fault-related features and reduce diagnostic reliability. Although many methods can achieve recognition accuracies close to 100% under ideal noise-free conditions [3], their performance usually declines markedly for noisy measurements. This indicates that mitigating noise-induced signal degradation and improving diagnostic robustness remain key challenges in the practical application of rolling bearing fault diagnosis.

Noise interference remains a major challenge in bearing fault diagnosis because it increases the difficulty of performing signal analysis and degrades diagnostic accuracy. Early studies mainly relied on conventional signal processing and handcrafted feature extraction methods [4]. Although effective in certain scenarios, such methods generally depend heavily on manual feature design and, thus, exhibit limited adaptability in the presence of nonstationary signals, transient noise, and complex interference. To address these limitations, the focus of research in this field has gradually shifted toward deep learning-based modelling paradigms. For instance, the SL Transformer proposed by Lee et al. captures long-range dependencies through self-attention, thereby enabling relatively robust feature extraction in noisy environments [5]; the WDCNN proposed by Zhang et al. enhances fault recognition from raw vibration signals through wide convolution and multi-scale feature learning [6]. Noise-robust modelling methods incorporating ensemble filtering, attention mechanisms, or multi-scale noise suppression have also been continually developed to improve feature representation and classification stability under complex interference conditions [7,8,9,10]. More broadly, intelligent learning-based methods have been increasingly explored in complex industrial systems to improve robustness and performance under conditions in which practical disturbances occur [11,12]. Overall, the mainstream modelling paradigm employed in existing studies still primarily centres on the original one-dimensional signal domain, while relatively few studies explicitly treat structured two-dimensional representations as a preceding representation domain for noise suppression and discriminative feature recovery. As a result, under strong noise conditions, the entanglement between fault-related structures and noise perturbations remains difficult to sufficiently unfold at the representation level, thereby limiting effective coordination between noise suppression and discriminative structure preservation.

Existing noise-robust fault diagnosis studies still largely rest on the premise that the original one-dimensional signal domain is sufficient to jointly accommodate noise suppression and fault identification. For bearing vibration signals, faults typically manifest as repetitive impulses, while real measurements are often corrupted by nonstationary modulation and random noise, causing fault-related structures and noise perturbations to become entangled in the original time domain [13,14]. Although deep models can improve robustness through end-to-end learning, they still act on one-dimensional temporal sequences whose structural organisation remains only partially unfolded. Consequently, under severe noise contamination, it is difficult to achieve effective denoising and discriminative structure preservation simultaneously. Moreover, even when two-dimensional transformed representations are introduced, they are mostly used as classification inputs rather than as an explicit preliminary domain for noise suppression and discriminative recovery [15,16]. In this sense, existing research efforts still focus primarily on enhancing network architectures within the original input domain, while the more fundamental question of whether a structured representation domain should first be constructed to better support the coordinated realisation of denoising and diagnosis remains insufficiently explored.

To address the above limitations, in this study, we propose a **P**hase-space **a**daptive **E**xpert **D**enoising **Net**work (PaEDNet), a robust fault diagnosis framework for rolling bearings under noisy conditions. Unlike approaches that mainly enhance model learning directly in the original one-dimensional signal domain, PaEDNet follows a representation-first strategy by first constructing a structured representation domain that is more suitable for the coordinated realisation of noise suppression and fault diagnosis. Specifically, the raw vibration signal is first transformed into a spatially organised two-dimensional representation, upon which structure restoration and downstream recognition are further performed, thereby forming a unified robust diagnostic pipeline.

Our main contributions in this study are threefold: First, this study moves beyond the conventional practice of performing noise-robust diagnosis directly in the original one-dimensional signal domain and proposes a structured-representation-oriented diagnostic paradigm for noisy bearing signals, in which the two-dimensional representation is elevated from a mere classification input to a preliminary restoration domain, thereby providing a new route for coordinating noise suppression and fault diagnosis. Second, to address the restoration problem in this representation domain, a CoPaMoE-augmented adaptive residual denoising mechanism is developed, enabling the network to produce more flexible restoration responses to different local patterns and, thus, better balance noise removal and discriminative structure preservation. Third, a unified robust diagnosis pipeline integrating representation construction, structure restoration, and downstream recognition is further constructed and validated. Experimental results under multiple signal-to-noise ratio settings show that the proposed framework achieves consistent superiority, especially stronger robustness in low-SNR scenarios.

## 2. Preliminaries

### 2.1. Problem Statement

In this study, fault diagnosis under noisy conditions is formulated not only as a perturbed-input classification problem but also as a representation modelling problem, namely how to unfold, restore, and discriminate fault-related structures under noise interference.

Let a clean vibration signal segment be denoted as

$$x_{i}=\left\{t_{i,j},t_{i,j+1},\ldots ,t_{i,j+n-1}\right\}\in R^{n},$$

with its corresponding class label $y_{i}\in \left\{1,2,\ldots ,C\right\}$. Under noisy conditions, the observed signal can be expressed as

$${\tilde{x}}_{i}=x_{i}+\eta_{i},$$

where $\eta_{i}$ denotes the noise component introduced via measurement degradation or external interference. Conventional models usually learn directly from ${\tilde{x}}_{i}$, and the corresponding optimisation objective can be written as

$$L_{\mathrm{noise}}=E_{\left(\tilde{x},y\right)}\left[\;l\left(g\left(f\left(\tilde{x}\right)\right),y\right)\;\right],$$

where f(·) denotes the feature extractor, g(·) denotes the classifier, and l(·) denotes the classification loss function. Therefore, the core problem addressed in this study is how to construct a representation domain that is more conducive to unfolding fault-related structures under strong noise conditions, as well as how to achieve the coordinated modelling of noise suppression and fault discrimination on that basis.

### 2.2. Background of Bearing Signal Analysis Under Noise

Since real industrial noise is usually affected by multiple factors, such as machine operation and environmental interference, it often exhibits multi-source coupling, nonstationarity, and poor reproducibility. Therefore, existing studies commonly construct controlled noisy measurement signals with predefined signal-to-noise ratio (SNR) levels to provide comparable and reproducible benchmarks for robustness evaluation [17,18]. In this setting, the core issue in bearing fault diagnosis is determining how to extract stable and discriminative fault representations from degraded measurement signals.

To address this issue, existing studies have mainly proceeded in two directions: representation transformation and denoising–diagnosis coordination. On one hand, related methods transform raw one-dimensional vibration sequences into more spatially organised two-dimensional representations through image encoding schemes such as Gramian Angular Fields, Gramian Angular Difference Fields, recurrence plots, and other variants, before performing condition identification using convolutional networks, attention mechanisms, or lightweight vision models [4,10,17,19,20,21,22]. On the other hand, some studies have further focused on the relationship between noise suppression and fault recognition, attempting to improve recognition stability under complex interference conditions through joint denoising and diagnosis modelling, multi-domain collaborative restoration, or noise-aware optimisation strategies [9,10,23,24]. However, these two lines of research remain partially disconnected. Most two-dimensional representation methods directly use transformed image-like representations as classification inputs, while relatively few further treat them as a preceding representation domain for noise suppression and discriminative structure recovery. Although denoising-related methods can reduce noise interference, their restoration objectives often do not sufficiently consider the differences between local textures, boundaries, and discriminative patterns in two-dimensional structural representations. Therefore, determining how to achieve more effective noise suppression and fault representation preservation within a structured representation domain remains an important problem for bearing fault diagnosis under noisy conditions. This also provides the direct motivation for the proposed PaEDNet.

## 3. Method

**Figure 1:**
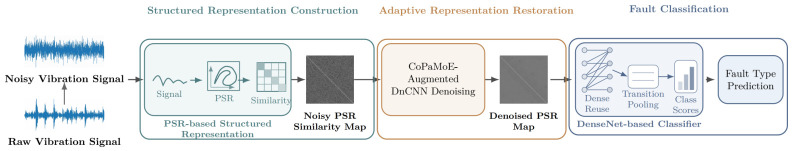
The overall framework of the proposed PaEDNet.

In this section, we present the proposed PaEDNet framework. To improve fault diagnosis under severe noisy conditions, the framework integrates phase-space-based representation construction, CoPaMoE-augmented adaptive restoration, and downstream fault identification into a unified pipeline. As illustrated in Figure 1, the overall workflow consists of three stages: structured representation construction, adaptive representation restoration, and fault classification. The following subsections describe each component in detail.

### 3.1. Phase-Space Representation Analysis

PaEDNet first applies phase-space reconstruction (PSR) to the raw vibration signal to construct a structurally separable pre-representation for subsequent two-dimensional denoising. Through delay embedding, PSR maps the time series into a higher-dimensional state space and unfolds the underlying dynamical relationships into geometric structures, thereby providing a more suitable basis for subsequent similarity representation and image-domain modelling.

Since the denoising target presented in this study is not the raw one-dimensional signal but the similarity map constructed from PSR, it is also necessary to analyse how additive noise propagates through the phase-space embedding and similarity mapping processes. This analysis is intended to characterise the statistical properties and spatial heterogeneity of noise in the resulting two-dimensional representation, as well as to provide a representation-level basis for the design of the subsequent input-adaptive denoising mechanism. For ease of derivation, a first-order local perturbation approximation is adopted in the following analysis.

To obtain a stable phase-space representation, the delay time τ and the embedding dimension *m* should be properly specified. In this study, the autocorrelation function (ACF) and false nearest neighbour (FNN) analyses are introduced as principled guidance for PSR parameter selection, so as to characterise the redundancy among delayed coordinates and the sufficiency of the embedding dimension. Specifically, the ACF is used to examine the decay of linear dependence across different delays, thereby providing guidance for the choice of τ. In practical terms, τ is typically selected near the first lag at which the autocorrelation decays to zero or a near-zero level, so as to reduce excessive linear redundancy between delayed coordinates while preserving useful dynamical dependence. The ACF is defined as

(1) $$R\left(\tau \right)=\frac{\sum_{t=1}^{N-\tau }\left(x\left(t\right)-\bar{x}\right)\left(x\left(t+\tau \right)-\bar{x}\right)}{\sum_{t=1}^{N}{\left(x\left(t\right)-\bar{x}\right)}^{2}},$$

where R(τ) denotes the autocorrelation value at delay τ, x(t) and x(t+τ) denote the sampled values of the vibration signal at time indices *t* and t+τ, respectively, $\bar{x}$ denotes the signal mean, and *N* denotes the signal length.

The embedding dimension *m* is determined using the false nearest neighbour (FNN) method. When *m* is too small, high-dimensional dynamical trajectories are prone to projection overlap in the low-dimensional space; as *m* increases, the proportion of false nearest neighbours gradually decreases and eventually stabilises, at which point the corresponding embedding dimension is considered a suitable choice. To quantify the variation in neighbourhood relationships before and after dimensional expansion, the following distance ratio is introduced:

(2) $$r_{ij}^{\left(m\right)}=\frac{{∥x_{i}^{\left(m+1\right)}-x_{j}^{\left(m+1\right)}∥}_{2}}{{∥x_{i}^{\left(m\right)}-x_{j}^{\left(m\right)}∥}_{2}},$$

where $x_{i}^{\left(m\right)}$ and $x_{j}^{\left(m\right)}$ denote reconstructed vectors in the *m*-dimensional phase space. In practical estimation, a neighbouring point is regarded as a false nearest neighbour if $r_{ij}^{\left(m\right)}$ exceeds a prescribed threshold, and the embedding dimension is then selected according to the stabilisation trend of the corresponding false-neighbour proportion.

Once τ and *m* are determined, the original time series {x(t)} is rearranged into matrix form as

(3) $$Y=\left[\begin{array}{cccc} x(1) & x(1+\tau ) & \cdots & x\left(1+(m-1)\tau \right)\\ x(2) & x(2+\tau ) & \cdots & x\left(2+(m-1)\tau \right)\\ \vdots & \vdots & \ddots & \vdots \\ x(T) & x\left(T+\tau \right) & \ldots & x\left(T+(m-1)\tau \right) \end{array}\right],$$

and the *t*-th phase-space vector is denoted as

(4) $$y_{t}={\left[x(t),x(t+\tau ),\ldots ,x(t+(m-1)\tau )\right]}^{⊤},\;t=1,2,\ldots ,T,$$

where

(5) T=N−(m−1)τ.

Using Equations (3)–(5), the nonlinear dynamical features and temporal dependencies present in the original signal are preserved within the geometric relationships among state vectors, enabling trajectory differences present across different operating conditions to be more fully unfolded.

To further exploit the structural information embedded in the phase space, the cosine similarity between phase-space vectors at different time steps is computed and mapped to a greyscale image. Assuming ${∥y_{i}∥}_{2}\neq 0$ and ${∥y_{j}∥}_{2}\neq 0$, the similarity matrix corresponding to the clean signal is defined as

(6) $$S_{ij}=\frac{y_{i}^{⊤}y_{j}}{{∥y_{i}∥}_{2}{∥y_{j}∥}_{2}},\;i,j=1,2,\ldots ,T,$$

which is further linearly mapped to the greyscale interval to yield the two-dimensional greyscale representation

(7) $$I_{ij}=\frac{S_{ij}+1}{2}.$$

In this way, the original one-dimensional vibration sequence is transformed into a two-dimensional structured image with spatial organisation, in which fault-relevant patterns primarily manifest as similarity stripes, localised block structures, and continuous textures, rather than as mere amplitude fluctuations along a single time axis.

Since signals acquired in practice typically contain noise, the input to the subsequent two-dimensional denoising module should be understood as a similarity map generated from a noisy signal. Accordingly, the propagation of noise through the phase-space embedding and similarity mapping processes is further analysed, beginning with the noisy signal model. Let the raw noisy vibration signal be expressed as

(8) $$\tilde{x}\left(t\right)=x\left(t\right)+n\left(t\right),$$

where x(t) is the ideal clean signal and n(t) is the additive noise term. Applying the same delay embedding map used for the clean signal to $\tilde{x}\left(t\right)$ yields the noisy phase-space vector

(9) $${\tilde{y}}_{t}={\left[\tilde{x}\left(t\right),\tilde{x}\left(t+\tau \right),\ldots ,\tilde{x}\left(t+\left(m-1\right)\tau \right)\right]}^{⊤},$$

which can be further written as

(10) $${\tilde{y}}_{t}=y_{t}+e_{t},$$

where

(11) $$e_{t}={\left[n(t),n(t+\tau ),\ldots ,n(t+(m-1)\tau )\right]}^{⊤}.$$

Using Equations (6) and (10), it is evident that scalar noise in the time domain, after undergoing phase-space embedding, no longer corresponds merely to amplitude perturbations at individual sample points; instead, it further affects the directional and distance relationships between state vectors.

Correspondingly, the similarity matrix generated from the noisy signal can be written as

(12) $${\tilde{S}}_{ij}=\frac{{\tilde{y}}_{i}^{⊤}{\tilde{y}}_{j}}{{∥{\tilde{y}}_{i}∥}_{2}{∥{\tilde{y}}_{j}∥}_{2}},$$

and its corresponding greyscale image is

(13) $${\tilde{I}}_{ij}=\frac{{\tilde{S}}_{ij}+1}{2}.$$

To analyse the propagation form of noise in the similarity map, define

(14) $$u_{i}=\frac{y_{i}}{∥y_{i}{∥}_{2}},\;u_{j}=\frac{y_{j}}{∥y_{j}{∥}_{2}},$$

so that

(15) $$S_{ij}=u_{i}^{⊤}u_{j}.$$

Here, $e_{i}$ and $e_{j}$ denote the embedded noise vectors corresponding to phase-space vectors $y_{i}$ and $y_{j}$, respectively, with both being specific instances of $e_{t}$ in Equation (10) at different time indices.

Under conditions in which locally small perturbations occur, a first-order approximation of Equation (12) yields

(16) $${\tilde{S}}_{ij}\approx S_{ij}+\Delta_{ij},$$

where $\Delta_{ij}$ denotes the perturbation introduced by noise at position (i,j) in the similarity map. Further denoting P as the identity matrix consistent with the dimension of the phase-space vectors, the first-order approximation takes the form

(17) $$\Delta_{ij}\approx \frac{u_{j}^{⊤}\left(P-u_{i}u_{i}^{⊤}\right)e_{i}}{∥y_{i}{∥}_{2}}+\frac{u_{i}^{⊤}\left(P-u_{j}u_{j}^{⊤}\right)e_{j}}{∥y_{j}{∥}_{2}}.$$

As shown in Equation (17), $\Delta_{ij}$ is jointly governed by the state direction, state energy, and projected perturbation of the embedded noise vector. Consequently, noise perturbations at different positions in the similarity map are not uniform additive terms, but they inherently exhibit position-dependence and structural dependence.

For analytical convenience, if one further adopts a local decorrelation approximation for the embedded perturbations and neglects the correlation induced by overlapping delay windows, the conditional variance of $\Delta_{ij}$ can be approximately expressed as

(18) $$\mathrm{Var}\left(\Delta_{ij}|y_{i},y_{j}\right)\approx \sigma_{e}^{2}\left(\frac{1-S_{ij}^{2}}{∥y_{i}{∥}_{2}^{2}}+\frac{1-S_{ij}^{2}}{∥y_{j}{∥}_{2}^{2}}\right),$$

where $\sigma_{e}^{2}$ denotes the equivalent variance scale of the embedded noise vector under this approximation. Since

(19) $${\tilde{I}}_{ij}-I_{ij}=\frac{{\tilde{S}}_{ij}-S_{ij}}{2}\approx \frac{\Delta_{ij}}{2},$$

the conditional variance in the greyscale image domain correspondingly satisfies

(20) $$\mathrm{Var}\left({\tilde{I}}_{ij}-I_{ij}|y_{i},y_{j}\right)\approx \frac{1}{4}\mathrm{Var}\left(\Delta_{ij}|y_{i},y_{j}\right).$$

These results indicate that the intensity of noise fluctuations varies across different positions in the similarity map, demonstrating that noise in this representation domain exhibits pronounced spatial heterogeneity.

On the other hand, since adjacent phase-space vectors are temporally continuous, the clean similarity matrix typically exhibits continuous structures near the diagonal neighbourhood, along with local stripe features and block-like texture patterns. To characterise this local continuity, the variation between adjacent state vectors is defined as

(21) $$d_{t}={∥y_{t+1}-y_{t}∥}_{2}.$$

When the signal evolves smoothly within a local region, $d_{t}$ changes gradually, indicating that the PSR similarity map preserves local continuity and structural organisation. In this representation domain, noise manifests as perturbations to continuous textures, local blocks, and boundary-like similarity patterns, rather than as simple point-wise amplitude disturbance in the original time series. Thus, denoising on the PSR similarity map is essentially a restoration of local structural consistency. This motivates the use of two-dimensional convolutional denoising, which can exploit local neighbourhood relationships in the similarity image. However, because such perturbations are position-dependent and structure-dependent, fixed denoising mapping is insufficient for diverse local regions, and an input-adaptive denoising module is required to balance noise suppression with fault-relevant structure preservation.

### 3.2. Adaptive Representation Restoration with CoPaMoE-Augmented DnCNN

**Figure 2:**
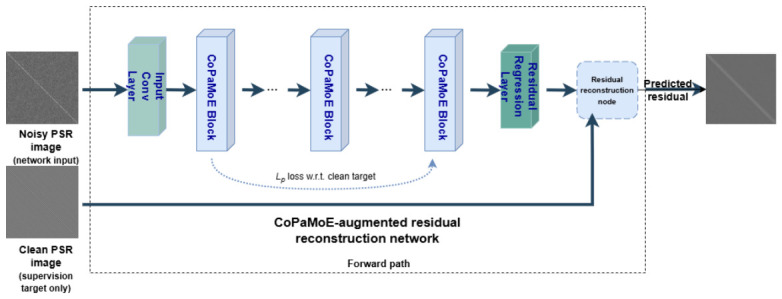
The architecture of the adaptive representation restoration stage with CoPaMoE-Augmented DnCNN.

Based on the PSR image-domain noise analysis in Section 3.1, this section introduces the adaptive representation restoration stage implemented via CoPaMoE-Augmented DnCNN. Specifically, DnCNN is adopted as the foundational residual denoising backbone, while the CoPaMoE mechanism is introduced to adaptively restructure its intermediate convolutional blocks for spatially heterogeneous noise restoration. The architecture of this stage is shown in Figure 2.

#### 3.2.1. Basic Residual Denoising Structure

In this study, we first adopt DnCNN as the two-dimensional denoising backbone. By learning residuals directly to predict the noise component rather than regressing the clean image, DnCNN can reduce the optimisation difficulty of denoising while maintaining the network’s expressive capacity [25]. For the PSR similarity maps constructed in this study, this modelling approach facilitates the decoupling of structural preservation and noise suppression.

Let $\tilde{I}$ denote the input noisy similarity map and $R(\tilde{I})$ denote the image-domain noise residual predicted via the network; then, the denoised output can be written as

(22) $$\hat{I}=\tilde{I}-R\left(\tilde{I}\right),$$

where $\hat{I}$ denotes the reconstructed denoised image.

The basic DnCNN consists of an input convolutional layer, a series of convolution–normalisation–activation units, and an output regression layer. Denoting the input feature of the *l*-th layer as $h_{l-1}$, the feature transformation can be expressed as

(23) $$h_{l}=\mathrm{ReLU}\;\left(\mathrm{BN}\;\left(W_{l}*h_{l-1}+b_{l}\right)\right),\;l=1,2,\ldots ,L,$$

where $h_{0}=\tilde{I}$, and $W_{l}$ and $b_{l}$ denote the convolutional kernel parameters and bias term of the *l*-th layer, respectively, with ∗ denoting the convolution operation.

During training, the basic residual denoising structure is optimised using a mean squared error loss. Let ${\tilde{I}}_{i}$ denote the noisy input image of the *i*-th training sample, and let its corresponding ground-truth noise residual be

(24) $$N_{i}={\tilde{I}}_{i}-I_{i},$$

where $I_{i}$ denotes the corresponding clean target image. The loss function is then defined as

(25) $$L_{\mathrm{MSE}}=\frac{1}{N}\sum_{i=1}^{N}{∥R\left({\tilde{I}}_{i}\right)-N_{i}∥}_{2}^{2},$$

#### 3.2.2. CoPaMoE Augmentation Mechanism

Although the basic DnCNN backbone provides a stable residual denoising structure, its shared convolutional kernels apply fixed mapping to the entire PSR similarity map. This is insufficient for spatially heterogeneous restoration, because different local regions exhibit different noise characteristics and preservation demands: continuous texture regions require the maintenance of structural continuity, whereas boundary- or stripe-like regions rely more heavily on retaining discriminative details. To address this limitation, the CoPaMoE mechanism is introduced to augment the intermediate convolutional blocks of DnCNN, thereby forming the CoPaMoE-Augmented DnCNN. By replacing the original fixed convolutional transformation with input-adaptive restoration mapping, the network can produce differentiated responses to different local noise patterns.

The MoE paradigm provides useful modelling inspiration for achieving this objective, since it can dynamically activate different sub-experts according to input features. However, directly inserting a conventional MoE block into DnCNN may introduce parameter redundancy and expert homogenisation [26,27,28]. Existing studies have shown that, without explicit mechanisms for expert differentiation, MoE models may degenerate into inefficient ensembles of redundant experts [29]. Meanwhile, the MPO parametrisation method proposed by Gao et al. demonstrates that efficient expert modelling can be achieved while preserving expressive capacity through core–auxiliary tensor decomposition [30]. Based on these observations, CoPaMoE jointly redesigns the parameter organisation of expert convolutional kernels and the corresponding dynamic routing mechanism around the restoration task on PSR similarity maps, so that the network retains a unified residual denoising backbone while acquiring region-adaptive restoration capability.

CoPaMoE operates on the intermediate convolutional blocks of the basic DnCNN, replacing the original standard shared convolutional mappings therein, while the input layer and the final residual regression layer retain the basic DnCNN structure. The *l*-th layer feature update in the original DnCNN takes the form

(26) $$h_{l}=\phi \;\left(W_{l}*h_{l-1}+b_{l}\right),\;\phi \left(\cdot \right)=\mathrm{ReLU}\left(\mathrm{BN}\left(\cdot \right)\right),$$

where $W_{l}$ is a fixed shared convolutional kernel. In this study, this fixed convolutional mapping is restructured into an adaptive convolutional operator $W_{l}\left(h_{l-1}\right)$ determined through the current input features, so that the *l*-th layer update is rewritten as

(27) $$h_{l}=\phi \;\left(W_{l}\left(h_{l-1}\right)*h_{l-1}+b_{l}\right).$$

As seen in Equations (26) and (27), the core modification in CoPaMoE-Augmented DnCNN involves replacing the fixed shared convolutional kernels in the intermediate convolutional blocks with input-conditioned adaptive convolutional mappings.

To construct this adaptive convolutional operator, the convolutional kernel $W_{l}$ of the *l*-th layer in Equation (26) is first reparametrised. For notational brevity, the convolutional weight of this layer is hereafter abbreviated as $W\in R^{I\times J}$, where $I=\prod_{k=1}^{m}i_{k},J=\prod_{k=1}^{m}j_{k}$, and $i_{k}$ and $j_{k}$ denote the factorised components of the input and output dimensions, respectively. *W* is further reshaped into its corresponding high-order tensor form, and the Matrix Product Operator (MPO) formalism is applied to parametrise this shared convolutional kernel, representing it as a set of fourth-order tensors ${\left\{T_{l}^{\left(k\right)}\right\}}_{k=1}^{m}$:

(28) $$T_{l}^{\left(k\right)}\in R^{d_{k-1}\times i_{k}\times j_{k}\times d_{k}},$$

where $d_{k}$ denotes the bond dimension, with $d_{0}=d_{m}=1$. Correspondingly, the shared convolutional weight of this layer can be expressed via tensor contraction as

(29) $$W\left(i_{1},\ldots ,i_{m};j_{1},\ldots ,j_{m}\right)=\sum_{d_{1},\ldots ,d_{m-1}}\prod_{k=1}^{m}T_{l}^{\left(k\right)}\left(d_{k-1},i_{k},j_{k},d_{k}\right).$$

The expression defined in Equation (29) constitutes the shared core convolutional representation across all experts, serving as the foundation for the common restoration backbone across different input patterns.

Building upon the shared core tensors ${\left\{T_{l}^{\left(k\right)}\right\}}_{k=1}^{m}$, in this study, we further introduce lightweight perturbation tensors ${\left\{A_{l,i}^{\left(k\right)}\right\}}_{k=1}^{m}$ for each expert *i* to construct the expert-specific convolutional kernel for this layer. Here, Contract(·) denotes tensor contraction along the MPO chain bond dimensions to recover the full convolutional kernel parameters for the corresponding expert. The convolutional kernel of the *i*-th expert can, therefore, be written as

(30) $$W_{l,i}=\mathrm{Contract}\left({\left\{T_{l}^{\left(k\right)}+A_{l,i}^{\left(k\right)}\right\}}_{k=1}^{m}\right).$$

As shown in Equation (30), the single shared convolutional kernel of this layer in the original DnCNN is extended into a set of expert convolutional kernels that share a common core but possess different personalised perturbations. In this way, while sharing the primary restoration capability, each expert can form differentiated responses in terms of local noise patterns, structural preservation strategies, and restoration tendencies.

Having obtained the expert convolutional kernels, the network further needs to adaptively determine the set of experts participating in mapping for the current layer based on the current input features. To this end, a routing module is designed to generate expert selection probabilities. Given the input feature map $h_{l-1}$ of the *l*-th layer,

(31) $$p_{l}={\mathrm{Router}}_{l}\left(h_{l-1}\right),\;p_{l}\in R^{M},$$

where *M* denotes the number of experts. The top-*k* experts with the highest probabilities are then selected to form the activation set $E_{l}\left(h_{l-1}\right)$, and the dynamic convolutional operator for this layer is expressed as

(32) $$W_{l}\left(h_{l-1}\right)=\sum_{i\in E_{l}\left(h_{l-1}\right)}p_{l,i}\;W_{l,i},$$

where $p_{l,i}$ denotes the routing weight of the *i*-th expert in the *l*-th layer. Correspondingly, each activated expert produces an intermediate convolutional output

(33) $$h_{l,i}=W_{l,i}*h_{l-1},$$

and the final output of this layer can be equivalently written as

(34) $$h_{l}=\mathrm{ReLU}\;\left(\mathrm{BN}\;\left(\sum_{i\in E_{l}\left(h_{l-1}\right)}p_{l,i}\cdot h_{l,i}+b_{l}\right)\right).$$

Equations (32)–(34) demonstrate that the restructured intermediate convolutional block can dynamically invoke different expert convolutional pathways based on the current input features, thereby transforming the originally static shared convolutional mapping into input-conditioned adaptive convolutional mapping.

To mitigate expert redundancy and mode collapse, a lightweight regularisation constraint is further imposed on the routing distribution of each CoPaMoE convolutional block. Denoting the routing distribution of the *l*-th CoPaMoE convolutional block as $p_{l}=\left(p_{l,1},\ldots ,p_{l,M}\right)$, its entropy is written as

(35) $$H\left(p_{l}\right)=-\sum_{i=1}^{M}p_{l,i}\log p_{l,i}.$$

During training, routing entropy regularisation is jointly optimised with the basic denoising objective, yielding the total loss function

(36) $$L=L_{\mathrm{MSE}}+\lambda_{\mathrm{ent}}L_{\mathrm{ent}},$$

where $\lambda_{\mathrm{ent}}$ is a balancing coefficient, $L_{\mathrm{MSE}}$ is defined by Equation (25), and

(37) $$L_{\mathrm{ent}}=-\frac{1}{N}\sum_{n=1}^{N}\sum_{l\in B}H\;\left(p_{l}^{\left(n\right)}\right),$$

with B denoting the set of all convolutional blocks in which CoPaMoE is introduced. This regularisation term constrains the routing distributions of different convolutional blocks, suppressing the rapid convergence of experts towards highly similar activation patterns in the early stages of training, thereby encouraging more stable functional differentiation among experts.

In summary, CoPaMoE is not a simple addendum to DnCNN, but rather a structural restructuring of its intermediate convolutional blocks tailored to the heterogeneous noise restoration requirements of PSR similarity maps. By transforming the fixed shared convolutional mapping into input-conditioned adaptive mapping jointly determined via shared cores, expert perturbations, and dynamic routing, the network acquires adaptive restoration capability oriented towards different local patterns, thereby facilitating the coordinated achievement of complex noise suppression and fault-relevant structure preservation.

### 3.3. Integrated Fault Diagnosis Framework

Building upon the above representation construction and adaptive denoising modules, PaEDNet performs downstream fault recognition on the restored representation, thereby completing the full diagnostic process from structured representation recovery to final fault identification.

**Figure 3:**
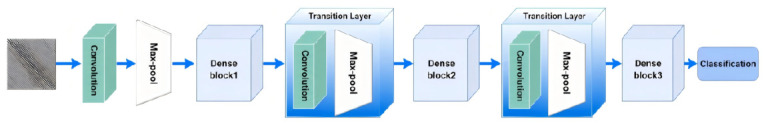
The architecture of the DenseNet-based downstream classification module in PaEDNet.

DenseNet is adopted as the downstream classifier because its dense connections promote multi-level feature reuse and fusion across depths. This enables the classifier to exploit local texture patterns, stripe-like similarity structures, and higher-level discriminative representations preserved in the restored PSR map. As illustrated in Figure 3, the classifier consists of an initial convolutional layer, multiple dense blocks, transition layers, and a final classification head, where the transition layer takes the form

(38) $$h^{\prime }=\mathrm{AvgPool}\left(\mathrm{Conv}2D(h)\right).$$

Denoting the image representation after denoising via CoPaMoE-Augmented DnCNN as $\hat{I}$, the classifier output can be expressed as

(39) $$z=f_{\mathrm{cls}}\left(\hat{I}\right),$$

where $f_{\mathrm{cls}}\left(\cdot \right)$ denotes the DenseNet classifier, and $z\in R^{C}$ denotes the predicted logits corresponding to *C* fault categories. After softmax normalisation, the class posterior probability is obtained as

(40) $$p\left(c|\hat{I}\right)=\frac{\exp(z_{c})}{\sum_{j=1}^{C}\exp\left(z_{j}\right)},\;c=1,2,\ldots ,C,$$

where $z_{c}$ denotes the output component corresponding to the *c*-th class. The final predicted class is then given by

(41) $$\hat{c}=\arg\max_{c}\;p\left(c|\hat{I}\right).$$

During training, the classification module is optimised using a cross-entropy loss. Let $c^{*}$ denote the ground-truth class label; the classification loss is defined as

(42) $$L_{\mathrm{cls}}=-\sum_{c=1}^{C}1\left(c=c^{*}\right)\log p\left(c|\hat{I}\right),$$

where 1(·) is the indicator function. In this way, the two-dimensional structural representation obtained after denoising directly participates in subsequent fault recognition, so that the efficacy of the denoising module is reflected not only in image restoration quality but also in the improvement in fault classification performance.

Accordingly, PaEDNet forms an integrated diagnostic pipeline from raw vibration signals to final fault labels. It should be noted that this integration is established at the inference level, whereas the denoising and classification modules are optimised in a stage-wise manner during training.

## 4. Experiments

### 4.1. Experimental Setup and Datasets

**Figure 4:**
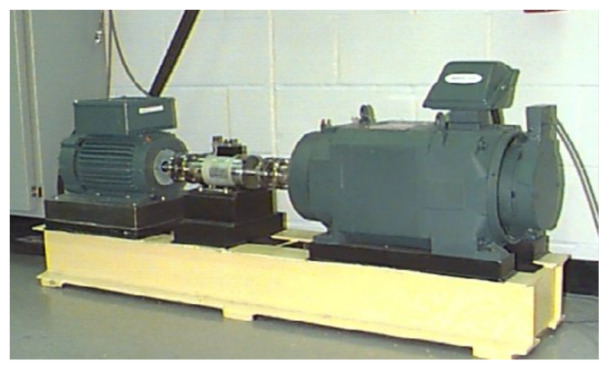
The CWRU bearing fault test rig.

**CWRU dataset.** The proposed method was first validated on the Case Western Reserve University (CWRU) bearing dataset [31]. Its fault test rig, shown in Figure 4, mainly consisted of a 2 hp motor, a torque transducer, and a power meter. Inner-race, outer-race, and rolling-element faults were introduced via electrical discharge machining (EDM). The dataset provided acceleration signals measured near both the drive end and the fan end. The drive-end fault data were sampled at 12 kHz and 48 kHz, while the fan-end fault data were sampled at 12 kHz. Fault diameters ranged from 0.007 to 0.040 inches, load conditions ranged from 0 to 3 hp, and shaft speed ranged approximately from 1797 to 1720 rpm. In the experiments, the raw vibration signals were segmented sequentially using a non-overlapping sliding window with a length of 1024, and the resulting samples were divided into training, validation, and test sets at a ratio of 7:1:2.

**Figure 5:**
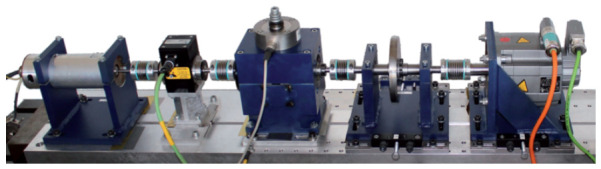
The PU bearing test platform.

**PU dataset.** Supplementary experiments were also conducted on the Paderborn University (PU) bearing dataset, whose test platform is shown in Figure 5. The dataset was obtained from the KAt-DataCenter, the official bearing data repository of the Chair of Design and Drive Technology at Paderborn University [32], and it contained synchronously acquired vibration and motor current signals covering healthy bearings and multiple real damaged states under four operating conditions [33]. For each setting, it provided 20 original recordings of 4 s sampled at 64 kHz. In this study, only the vibration signals were used, and a three-class cross-condition task was constructed by grouping all samples into healthy, outer-race fault, and inner-race fault categories. The raw signals were segmented using a sliding window with a length of 2048. To avoid overlap between different subsets at the original recording level, the first 19 recordings under each setting were used to construct the training and validation sets, while the remaining one was reserved for testing.

For robustness evaluation, AWGN was added to the original one-dimensional vibration signals of both datasets to generate controlled noisy samples at different SNR levels. In phase-space reconstruction, the time delay and embedding dimension were fixed at τ=7 and m=20, respectively, across all datasets and noise settings. These values were determined from preliminary ACF and FNN analyses on representative clean training samples and then kept fixed throughout all experiments to ensure a consistent representation construction protocol and fair robustness comparison across different datasets and SNR conditions. In the CoPaMoE module, three experts were used with a top-2 routing strategy, and four intermediate convolutional blocks of the denoising network were replaced by CoPaMoE blocks. The model was optimised using AdamW with an initial learning rate of $5\times 10^{-4}$. All experiments were repeated three times with different random seeds, and the reported results are given as the average over the three runs.

### 4.2. Comparison Experiments

To systematically evaluate the robustness of the proposed PaEDNet under different noise level conditions, comparative experiments were conducted using four SNR settings, namely 0 dB, −2 dB, −4 dB, and −6 dB. The compared methods were all representative bearing fault diagnosis models designed for noisy environments, covering different technical routes such as convolutional feature learning, sequence modelling, attention enhancement, multi-scale anti-noise modelling, and joint denoising–diagnosis frameworks. Specifically, the compared models included WDCNN [6], CNN–LSTM [34], ResNet [35], EfficientNet/CWT-AttentionEfficientNet [8], SC-CAPSENET [7], SL Transformer [5], MLSCA [17], and MDCAE-CACNN [10]. The quantitative results on the CWRU and PU datasets are summarised in Table 1 and Table 2, respectively.

Overall, all compared methods show performance degradation as the noise intensity increases, although the extent of degradation differs across models. For the CWRU dataset, while MDCAE-CACNN achieved the highest accuracy at 0 dB (99.83%), its performance declined more rapidly under severe noise conditions, decreasing to 89.69% at −6 dB, whereas PaEDNet still maintained an accuracy of 93.98%. A similar tendency was observed for MLSCA, whose accuracy decreased from 96.63% to 90.13% over the same SNR range. For the PU dataset, PaEDNet consistently achieved the best results across all four SNR conditions, and under the most challenging condition, i.e., −6 dB, it still clearly outperformed MLSCA (85.91%) and MDCAE-CACNN (82.73%). These results demonstrate the superior robustness and stability of the proposed framework under complex noise conditions.

**Figure 6:**
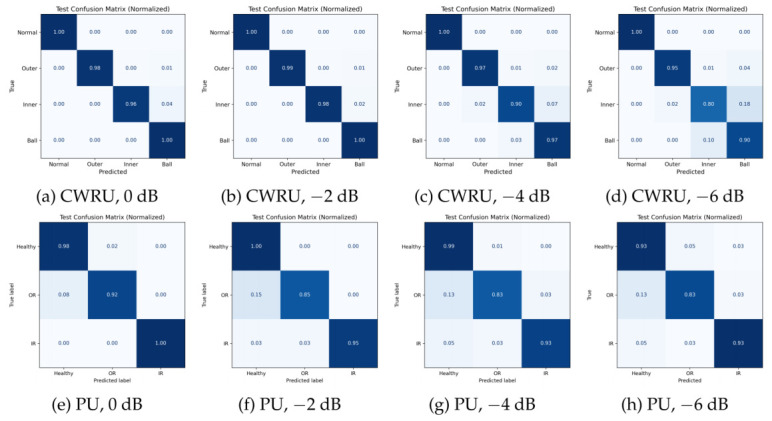
The normalised confusion matrices of PaEDNet on the CWRU and PU datasets at different SNR levels.

To further provide a class-wise view of the recognition results, Figure 6 presents the normalised confusion matrices of PaEDNet on the CWRU and PU datasets at different SNR levels, showing that the proposed method generally maintains clear diagonal dominance even under low-SNR conditions.

### 4.3. Overall Ablation Study

To verify the contribution of each major component, an overall ablation study was conducted on both the CWRU and PU datasets, and the results are reported in Table 3 and Table 4, respectively. Specifically, Signal-Based Baseline performs fault classification directly on the original one-dimensional vibration signals, PSR Representation introduces the proposed phase-space-based two-dimensional representation, PSR + Standard Denoising further adds a standard denoising module in this representation domain, and PaEDNet denotes the complete framework proposed in this study. As shown in Table 3 and Table 4, a consistent progressive improvement trend can be observed on both datasets, from Signal-Based Baseline to PSR Representation, then to PSR + Standard Denoising, and finally to PaEDNet. First, the improvement from Signal-Based Baseline to PSR Representation indicates that the structured two-dimensional representation helps enhance the separability between fault-related structures and noise disturbances under noisy conditions. Second, the further gain achieved by PSR + Standard Denoising suggests that the PSR-based representation serves not only as a classification input but also as an effective preliminary domain for noise suppression and structure recovery. Finally, PaEDNet consistently achieves the best results across all SNR settings, indicating that fixed standard denoising mapping is still insufficient in this representation domain, whereas the proposed adaptive restoration mechanism can better balance noise suppression and discriminative structure preservation.

This advantage becomes more evident under severe noise conditions. For example, under the −6 dB condition, PaEDNet achieves 93.98% on the CWRU dataset, outperforming PSR + Standard Denoising (89.65%), and achieves 90.45% on the PU dataset, which is also higher than the 87.88% for PSR + Standard Denoising. Overall, these results show that the performance improvement for the proposed framework comes not only from the downstream classifier itself but also from the coordinated effects of structured representation construction, representation domain denoising, and adaptive restoration, thereby enabling more robust fault diagnosis under complex noise conditions.

### 4.4. Ablation Study of the CoPaMoE Mechanism

To validate the effectiveness of the CoPaMoE mechanism, ablation experiments were conducted on both the CWRU and PU datasets, as reported in Table 5 and Table 6. The results show that the complete PaEDNet consistently achieves the best performance at all noise levels. In contrast, replacing the dynamic expert structure with static convolution leads to the most significant decline in performance, indicating that input-conditioned expert selection is a key factor for improving robustness. Removing the expert-specific perturbation mechanism or the router entropy constraint also degrades performance, further suggesting that the effectiveness of CoPaMoE depends not only on dynamic routing but also on the parametrised differentiation among experts.

**Figure 7:**
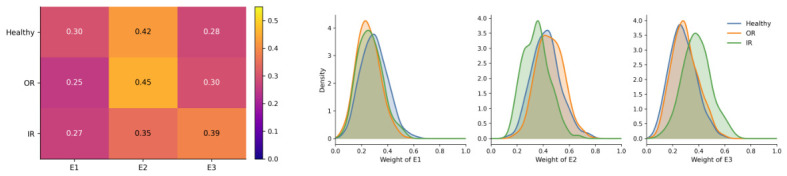
The visualisation of expert-routing behaviour on the PU dataset. (**Left**): Average expert weights for different fault categories. (**Right**): Class-wise density distributions of expert weights for E1, E2, and E3.

Furthermore, Figure 7 presents the average expert weights and their distributions for different fault categories on the PU dataset. Different categories exhibit distinct expert-usage tendencies: healthy and OR samples assign relatively higher weights to E2, while IR samples show a comparatively stronger response for E3. This result indicates that the routing mechanism can learn fault-related expert allocation patterns, thereby providing mechanistic support for the performance gains of CoPaMoE.

### 4.5. Performance Investigation of Denoising Backbones

**Figure 8:**
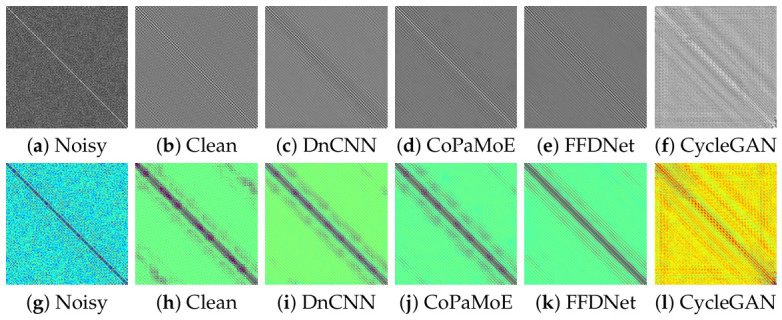
The visual comparison of denoising results under −6 dB noise conditions for different models. The first row shows greyscale visualisations, and the second row shows the corresponding jet-colourmap visualisations.

To compare the restoration capability of different denoising backbones under severe noise conditions, three representative denoising methods, namely CycleGAN [36], FFDNet [37], and DnCNN, are evaluated together with the proposed CoPaMoE under the −6 dB condition, as shown in Figure 8. The results show that CoPaMoE achieves the best performance in both visual quality and quantitative evaluation, with a PSNR of 15.7 dB, which is higher than those of DnCNN (13.5 dB), FFDNet (12.4 dB), and CycleGAN (11.3 dB). Further visual inspection indicates that CoPaMoE restores the main diagonal structure and local texture patterns more clearly while suppressing background noise more effectively, demonstrating that it is more suitable as the core denoising module in PaEDNet.

## 5. Conclusions

PaEDNet is proposed as a robust fault diagnosis framework for rolling bearing vibration signals under severe noisy conditions. By constructing a structured two-dimensional similarity representation through phase-space reconstruction and performing adaptive restoration and downstream fault recognition within this representation domain, the proposed method integrates representation construction, denoising, and diagnosis into a unified robust diagnostic pipeline.

The ablation studies and expert-routing analyses further confirm that the performance gains come not only from the downstream classifier but also from the coordinated effects of structured representation construction, adaptive expert restoration, and routing design. Overall, in this study, we suggest that robust fault diagnosis in noisy environments depends not only on the discriminative capability of the classifier but also on whether fault-related structures can be more effectively unfolded and restored before recognition. In future research, we will extend the proposed framework to more complex operating conditions and real industrial scenarios to further improve its generalisation ability and practical applicability.

## 6. Patents

The authors declare that no patents have resulted from the study reported in this manuscript.

## Figures and Tables

**Table 1:** Model performance on the CWRU dataset at different SNR levels.

Model	0 dB	−2 dB	−4 dB	−6 dB
WDCNN	81.92 ± 0.91	67.38 ± 1.18	57.45 ± 1.46	50.13 ± 1.87
CNN–LSTM	68.99 ± 1.12	59.33 ± 1.43	37.99 ± 1.96	30.73 ± 2.31
ResNet	68.33 ± 1.05	58.99 ± 1.38	33.33 ± 2.08	30.21 ± 2.42
CWT-AttentionEfficientNet	93.11 ± 0.46	90.41 ± 0.58	89.86 ± 0.67	87.87 ± 0.84
SC-CAPSENET	92.54 ± 0.55	83.78 ± 0.83	71.63 ± 1.17	65.47 ± 1.54
SL Transformer	92.44 ± 0.39	89.95 ± 0.51	85.55 ± 0.72	83.37 ± 0.96
MLSCA	96.63 ± 0.24	95.36 ± 0.31	92.86 ± 0.47	90.13 ± 0.63
MDCAE-CACNN	**99.83 ± 0.08**	98.83 ± 0.15	91.67 ± 0.42	89.69 ± 0.71
PaEDNet	99.64 ± 0.11	**99.03 ± 0.16**	**96.12 ± 0.29**	**93.98 ± 0.43**

**Note**: Bold values indicate the best performance among all compared methods under corresponding SNR conditions.

**Table 2:** Model performance on the PU dataset under different SNR levels.

Model	0 dB	−2 dB	−4 dB	−6 dB
WDCNN	76.81 ± 1.12	78.63 ± 1.24	72.72 ± 1.53	59.54 ± 1.98
CNN–LSTM	91.36 ± 0.74	81.81 ± 1.03	82.31 ± 1.12	78.63 ± 1.47
ResNet	90.90 ± 0.58	90.90 ± 0.66	86.78 ± 0.81	84.75 ± 1.03
CWT-AttentionEfficientNet	90.26 ± 0.63	84.22 ± 0.79	82.18 ± 0.91	78.64 ± 1.12
SC-CAPSENET	90.45 ± 0.67	86.82 ± 0.82	79.09 ± 1.08	79.09 ± 1.19
SL Transformer	90.00 ± 0.49	88.64 ± 0.57	83.18 ± 0.76	81.82 ± 0.95
MLSCA	92.73 ± 0.34	91.36 ± 0.42	86.82 ± 0.61	85.91 ± 0.74
MDCAE-CACNN	92.27 ± 0.29	92.27 ± 0.36	88.64 ± 0.55	82.73 ± 0.81
PaEDNet	**96.81 ± 0.18**	**95.45 ± 0.24**	**90.45 ± 0.33**	**90.45 ± 0.41**

**Note**: Bold values indicate the best performance among all compared methods under corresponding SNR conditions.

**Table 3:** The results for the ablation study conducted using the CWRU dataset at different SNR levels.

Variant	0 dB	−2 dB	−4 dB	−6 dB
Signal-Based Baseline	82.92±0.84	75.40±1.36	73.60±1.51	71.67±1.88
PSR Representation	90.28±0.42	88.35±0.51	85.34±0.63	85.23±0.71
PSR + Standard Denoising	95.28±0.24	95.12±0.29	93.13±0.37	89.65±0.48
PaEDNet	99.64±0.11	99.03±0.16	96.12±0.29	93.98±0.43

**Note**: Bold values indicate the best performance among all compared methods under corresponding SNR conditions.

**Table 4:** The results for the ablation study conducted using the PU dataset at different SNR levels.

Variant	0 dB	−2 dB	−4 dB	−6 dB
Signal-Based Baseline	90.90±0.73	90.00±0.81	84.00±0.96	80.00±1.14
PSR Representation	92.31±0.38	91.78±0.44	86.24±0.57	83.26±0.69
PSR + Standard Denoising	94.24±0.26	93.21±0.32	88.32±0.41	87.88±0.52
PaEDNet	96.81±0.18	95.45±0.24	90.45±0.33	90.45±0.41

**Note**: Bold values indicate the best performance among all compared methods under corresponding SNR conditions.

**Table 5:** An ablation study of the CoPaMoE mechanism conducted using the CWRU dataset at different SNR levels.

Model	0 dB	−2 dB	−4 dB	−6 dB
PaEDNet	99.64±0.11	99.03±0.16	96.12±0.29	93.98±0.43
PaEDNet-StaticConv	96.87±0.21	94.22±0.28	88.54±0.41	82.37±0.56
PaEDNet-w/o Expert Perturbation	98.41±0.17	96.67±0.23	91.78±0.35	87.22±0.49
PaEDNet-w/o Router Entropy	98.92±0.14	97.45±0.20	94.39±0.31	91.13±0.45

**Table 6:** An ablation study of the CoPaMoE mechanism conducted using the PU dataset at different SNR levels.

Model	0 dB	−2 dB	−4 dB	−6 dB
PaEDNet	96.81±0.18	95.45±0.24	90.45±0.33	90.45±0.41
PaEDNet-StaticConv	94.03±0.29	92.72±0.36	85.56±0.52	86.64±0.61
PaEDNet-w/o Expert Perturbation	95.42±0.24	93.26±0.31	87.67±0.46	86.31±0.57
PaEDNet-w/o Router Entropy	96.10±0.21	94.12±0.28	88.85±0.39	88.62±0.48

## Data Availability

The raw data used in this study are publicly available from the CWRU and PU benchmark datasets. The processed data generated in this study will be made available by the corresponding author upon reasonable request.
